# Physiological- and performance-related effects of acute olive oil supplementation at moderate exercise intensity

**DOI:** 10.1186/s12970-019-0279-6

**Published:** 2019-03-01

**Authors:** Laura Esquius, Sergi Garcia-Retortillo, Natàlia Balagué, Robert Hristovski, Casimiro Javierre

**Affiliations:** 1 FoodLab, Faculty of Health Sciences, Universitat Oberta de Catalunya, Avda. del Tibidabo, 39-43, 08035 Barcelona, Spain; 2 Department of Physiological Sciences, School of Medicine, Universitat de Barcelona (UB), C. Feixa Llarga, s/n, Hospitalet de Llobregat, 08907 Spain; 3 Complex Systems in Sport, University School of Health and Sport (EUSES), University of Girona, C. Francesc Macià 65, 17190 Salt, Spain; 4 Complex Systems in Sport, Institut Nacional d’Educació Física de Catalunya (INEFC), Universitat de Barcelona, Avda. de l’Estadi 12-22, 08038 Barcelona, Spain; 5 Faculty of Physical Education, Sport and Health, Ss Cyril and Methodius University of Skopje, Zeleznicka BB, 1000 Skopje, Republic of Macedonia

**Keywords:** Cardiorespiratory coordination, Olive oil, Polyphenols, Unsaturated fatty acids, Dietary supplementation, Cardiorespiratory exercise testing, Complex adaptive systems, Principal components analysis

## Abstract

**Background:**

The consumption of olive oil is associated with a diminished risk of cardiovascular disorders and mortality, but the impact of olive oil supplementation on endurance performance is still unclear. Since the beneficial effects of olive oil are observed at a systemic level, its effectiveness may not be precisely measured through the commonly registered maximal and threshold values of some physiological and performance parameters. In contrast, we suggest evaluating it through variables able to capture the coordinated behaviour of physiological systems. Thus, the aim of the current research was to assess the effect of an acute extra virgin olive oil supplementation on cardiorespiratory coordination (CRC) and performance, compared to palm oil.

**Methods:**

Three separate effort test sessions were carried out separated by 7-day interval. During each session, participants (*n* = 7) repeated the same progressive and maximal walking test, but under different dietary supplementations in a randomized order: (1) olive oil, (2) palm oil, and (3) placebo. A principal component (PC) analysis of selected cardiovascular and cardiorespiratory variables was carried out to evaluate CRC. Eigenvalues of the first PC (PC_1_) and the loadings of the cardiorespiratory variables onto PC_1_ were compared among dietary supplementations. In order to more accurately evaluate CRC, all the tests were divided into 3 equal sections, corresponding to low, moderate, and high exercise intensities, and the aforementioned procedure was repeated for each section in all the tests.

**Results:**

Statistically significant differences were observed regarding PC_1_ eigenvalues among dietary supplementations (*χ*^*2*^ (8,2) = 6.3; *p* = .04), only at moderate intensity exercise. Specifically, PC_1_ eigenvalues were higher under olive oil compared to palm oil (2.63 ± 0.51 vs. 2.30 ± 0.28; *Z* = 2.03; *p* = .04; *d =* 0.80) and placebo supplementations (2.63 ± 0.51 vs. 2.38 ± 0.36; *Z* = 2.20; *p* = .03; *d =* 0.57).

**Conclusions:**

Supplementation with extra virgin olive oil increased CRC during a progressive walking test at moderate intensity, although did not change performance and other physiological markers. CRC analysis appears as a sensitive tool to investigate the physiological and performance effects of dietary supplementations.

## Introduction

Olive oil is the main cornerstone of the Mediterranean diet and its consumption, specifically the extra virgin variety, is associated with a reduced inflammation and a diminished risk of cardiovascular disorders and mortality [1–3]. These benefits may be related to its source of polyphenols, which have been shown to possess antimicrobial, antioxidant and anti-inflammatory systemic properties [4, 5]. Despite the large amount of beneficial effects on health, the impact of olive oil supplementation on endurance performance is still unclear [6]. These effects have been measured through specific aerobic physiological markers like maximal oxygen uptake (VO_2max_), but not according to variables able to capture the dynamic interactions among physiological systems.

On the basis that diets high in unsaturated fatty acids and endurance exercise share different positive effects on metabolic and cardiovascular health, and given that both seem to increase fat oxidative capacity, earlier research hypothesised that their combination may have synergic effects [7]. The main results revealed that unsaturated fatty acids supplementation tended to slightly increase fat oxidation after training compared to control conditions. However, these changes were not reflected in VO_2max_ or in other performance and physiological parameters [6–8]. Moreover, regarding the antioxidant effect of beverages containing polyphenols on physical performance and physiological markers, the results are not yet entirely clear, with studies reporting controversial effects [9]. These unclear results suggest that VO_2max_ and other commonly registered physiological and performance variables might not be sensitive enough to detect specific exercise-related changes as has been suggested by other authors [10–12]. A feasible explanation for the lack of sensitivity may stem from the fact that they provide little information on the nature of the dynamic interactions between physiological systems and their common role in an integrated network [13, 14]. More specifically, cardiovascular and respiratory systems change their interaction as a consequence of exercise [10]. Therefore, to capture such specific interactions, the time series analysis and the detection of coordinative variables [15] seems a recommendable strategy. These research approaches can detect not only quantitative differences related to maximal physiological values (e.g., VO_2max_), but also qualitative changes related to the coordinated activity among physiological systems, and their changes under exercise-related constraints [16, 17]. This coordinative changes occurring at systemic level as a consequence of exercise, although much less studied than those occurring at cellular or subcellular level, are not less relevant.

Since the anti-inflammatory and antioxidant effects of olive oil are observed at a systemic level [5], and its effectiveness may not be precisely measured through the commonly registered physiological and performance parameters, we proposed here to use a recently investigated coordinative variable (cardiorespiratory coordination; CRC) which has shown a higher responsiveness to training [10] and workload accumulation [11], in contrast to VO_2max_ and other markers of aerobic fitness. CRC is a novel variable informing about the co-variation of cardiovascular and respiratory variables during cardiorespiratory exercise testing [10, 11]. It is estimated through principal components analysis (PCA), performed on the time series of selected variables. In contrast to isolated cardiorespiratory outcomes, the cardiorespiratory response to exercise can be represented through PCA, i.e., by a short set of principal components (PCs) extracted in decreasing order of importance (the first PC accounting for most of the variation). Principal components represent the maximum possible fraction of the variability from the original data, so that the total number of PCs reflects the degree of coordination among the selected variables. As pointed out by Kelso [18], a dimension reduction is a hallmark of formation of coordinative structures, and so, the decrease in the number of PCs and/or the increase in PC eigenvalues can be interpreted as an improvement in the efficiency of CRC (see Balagué et al. [10] for a detailed explanation).

Accordingly, the aim of the current research was to assess the effect of an acute fatty acid supplementation, in the form of extra virgin olive oil rich in polyphenols, on CRC and physiological and performance variables, compared to palm oil rich in unsaturated fatty acids and without polyphenols. We hypothesized that the positive effects of olive oil supplementation would be mainly reflected on CRC.

## Material and methods

### Participants

To determine the sample size for this study a power analysis was conducted using G*Power 3.1. [19]. Similar studies of CRC during exercise [11] have reported moderate and large effect sizes. Using an effect size of *d* = 0.75, *α* < .05, power (1—*β*) = .95, a minimum sample size of seven participants was established. Thus, seven healthy males (age 32.2 ± 4.3; height 180 ± 5.4 cm; body mass 74.9 ± 7.7 Kg; body mass index 23.1 ± 1.7 Kg.m^− 2^), who were active runners competing at national level for at least 5 years and engaged in endurance activities between four and seven times per week (10-14 h), volunteered to participate in the study. Exclusion criteria consisted of: a) current or previous injury against exercise testing, taking fish oil or other fatty acids supplements, and c) any other conditions that may prevent the performance of a maximal exercise protocol. The experiment was approved by the local ethical committee and carried out according to the Helsinki Declaration. Participants read the study’s description and risks and signed an informed consent before taking part in the study.

### Intervention and procedure

#### Study design

In this randomized cross-over controlled double-blind trial, three separate effort test sessions were carried out separated by 7-day intervals. During each session, participants repeated the same maximal walking test, but under different supplementations: (1) olive oil, (2) palm oil, and (3) placebo. Supplementation in the form of a gel at a temperature of about 10 °C was orally administered blind to each participant. The testing order was randomized using a random number generator. On all visits participants reported to the laboratory in a similar postprandial state (3 h after a light meal), and were instructed not to perform any vigorous physical activity for 72 h before testing. The ratio of the light meal was approximately 70% carbohydrates, 15% lipids, and 15% proteins. The following breakfast suggestion was provided: orange juice (250 g), whole milk (250 g), cereals (70 g). Participants were requested to eat the same light meal before each test session and not change the diet during the study period.

#### Maximal walking test

A maximal walking test ensuring high overall energy cost, and lasting longer time on Fat_max_ zone was selected. The test (Quasar, HP cosmos sports & medical gmbh, Nussdorf-Traunstein, Germany) was conducted at a constant velocity of 6.5 Km/h and the slope was increased by 1% every 4 min. Once the treadmill slope reached 20%, the velocity was increased by 1 Km/h every 4 min until volitional exhaustion. During the test, participants breathed through a valve (Hans Rudolph, 2700, Kansas City, MO, USA) and respiratory gas exchange was determined through an automated open-circuit device (Metasys, Brainware, La Valette, France). Oxygen and CO_2_ content and air flow rate were monitored every 30 s. Prior to each test, the system was calibrated with the following mixture: O_2_ 15%, CO_2_ 5%, N_2_ balanced (Carburos Metálicos, Barcelona, Spain), as well as with ambient air. Electrocardiogram (ECG) was continually recorded (CardioScan v.4.0, DM Software, Stateline, Nevada, USA) to monitor heart rate (HR). All tests were carried out in a well-ventilated lab; the place temperature was 23 °C and the relative humidity 48%, with changes of no more than 1 °C in temperature and 10% in relative humidity.

#### Supplements

The active and placebo supplements were prepared using orange juice, with added modified starch to achieve a gel texture; the active A supplement also included extra virgin olive oil, and the Active B also included palm oil (see Table 1). Orange juice was used to obtain a nice taste in all the supplements, and to mask the taste of both olive and palm oil actives, achieving the same taste for all the supplements. The calorific value and fat content of the active supplements were much higher than in the placebo. The supplement was taken in the lab, 1 h before the beginning of the test. A 25 ml dose of extra virgin olive oil was selected since it does not significantly induce a postprandial lipemia or an increase in oxidation markers in vivo, while a dose of 50 ml induces it [20].

**Table 1:** Supplement ingredients and nutritional content

	Active A	Active B	Placebo
Ingredients	100 ml orange juice	100 ml orange juice	100 ml orange juice
	25 ml Extra-virgin olive oil	25 ml Palm oil	8 g Modified starch
	8 g Modified starch	8 g Modified starch	
Energy (Kcal)	277	277	52.8
Lipids (g)	25	25	0.1
Carbohydrates (g)	12.6	12.6	12.6

### Data analysis

#### Cardiorespiratory coordination

To study CRC, a principal components analysis (PCA) was carried out on the time series of selected cardiorespiratory variables in all tests and participants: expired fraction of O_2_ (FeO_2_), expired fraction of CO_2_ (FeCO_2_), ventilation (VE), and HR. Oxygen pulse, oxygen consumption, respiratory equivalents, respiratory exchange ratio, and other frequently recorded parameters during cardiorespiratory testing were not included in the PCA given their known deterministic mathematical relation with the aforesaid variables [11]. Continuous blood pressure monitoring could not be provided in this study. However, non-published results of our lab have shown similar results while analysing CRC with and without continuous blood pressure measurement. Bartlett’s sphericity test and the KMO (Kaiser-Mayer-Olkin [21]) index were computed in order to evaluate the suitability of the application of the PCA. The number of PCs was defined by the Kaiser-Gutmann criterion, which recognizes PCs with eigenvalues λ ≥ 1.00 as a significant [22]. Eigenvalues of the first PC (PC_1_) and the loadings of selected cardiorespiratory variables onto PC_1_ were compared among dietary supplementations by means of a Freedman ANOVA test and a Wilcoxon matched pairs test, since PC_1_ contains the largest amount of the data variance. Statistical analyses were completed using SPSS (v.23, SPSS Inc., USA).

In order to more accurately study CRC, all tests were divided into three equal time sections (i.e., total exercising time / 3). The first, second, and third sections corresponded to low, moderate, and high exercise intensity, respectively. The cutting points between low to moderate, and moderate to high intensities were located at 40.29 + 10.57 and 70.81 + 7.08% VO_2peak_, respectively. The aforementioned procedure was repeated for each section in all the tests. The total number of PCs and PC_1_ eigenvalues reflects the dimensionality or degree of cardiorespiratory coordination (higher numbers of PCs and/or lower PC_1_ eigenvalues entail lower coordination, whereas lower numbers of PCs and/or higher PC_1_ eigenvalues imply higher coordination (for a detailed explanation see Balagué et al. [10] and Garcia-Retortillo et al.[11]).

#### Physiological and performance variables

Performance (measured through the exercising time) and maximal and threshold values of cardiorespiratory variables were recorded during each test. Aerobic (AT) and anaerobic (AnT) thresholds were obtained through O_2_ and CO_2_ respiratory equivalents method [23]. A repeated measures ANOVA with Bonferroni post-hoc test was used to compare values at AT, AnT, and maximal performance among dietary supplementations. Effect sizes (Cohen’s *d*) were computed to demonstrate the magnitude of standardized mean differences and an alpha level was set at 0.05 for all statistical tests.

## Results

### Cardiorespiratory coordination

Bartlett’s sphericity test (*p* < .001) and the KMO index (0.70 + 0.14) showed a good sampling adequacy. At low intensity, all participants showed 2 PCs in the three dietary supplementations. While PC_1_ was formed by VE, FeCO_2_, and HR, PC_2_ was mainly formed by FeO_2_ (see Table 2). No significant differences were found in PC_1_ eigenvalues among dietary supplementations at low intensity. Similar to the results at low intensity, at moderate intensity all participants showed 2 PCs in the three supplementations. However, Friedman ANOVA showed statistically significant differences regarding PC_1_ eigenvalues among dietary interventions (*χ*^*2*^ (8,2) = 7; *p* = .03; see Table 2). Specifically, olive oil supplementation revealed higher PC_1_ eigenvalues compared to palm oil (*Z* = 2.03; *p* = .04; *d =* 0.80) and placebo supplementations (*Z* = 2.20; *p* = .03; *d =* 0.57), indicating more co-variation among selected cardiorespiratory variables at moderate intensity. Significant differences between dietary supplementations were only found in the VE projection (*χ*^*2*^ (8,2) = 6.3; *p* = .04; see Table 2). Concretely, VE projection was significantly higher in olive oil supplementation compared to palm oil (Z = 2.37; *p* = .02; *d* = 4.71), but not compared to placebo (*Z* = 0.56; *p* = .60). No significant differences were found in PC_1_ eigenvalues between palm oil and placebo supplementations at moderate exercise intensity. Finally, all participants showed 2 PCs at high intensity, whereas PC_1_ was loaded by VE, FeO_2_, and HR, PC_2_ was mainly formed by FeCO_2_. No significant differences were found in PC_1_ eigenvalues among dietary supplementations at high intensity.

**Table 2:** Means (standard deviations) of PC_1_ eigenvalues and projection of the selected cardiorespiratory variables onto PC_1_

	1st third			2nd third			3rd third		
	Olive Oil	Palm Oil	Placebo	Olive Oil	Palm Oil	Placebo	Olive Oil	Palm Oil	Placebo
PC_1_ Eigenvalues	2,47	2,43	2,53	2,63^a^	2,30^a^	2,38^a^	2,48	2,42	2,59
	(0,42)	(0,58)	(0,26)	(0,51)	(0,28)	(0,36)	(0,39)	(0,32)	(0,21)
Veprojection	0,86	0,82	0,84	0,91^a^	0,79^a^	0,89^a^	0,83	0,92	0,96
	(0,06)	(0,13)	(0,07)	(0,03)	(0,17)	(0,05)	(0,34)	(0,06)	(0,02)
FeO_2_projection	0,57	0,67	0,58	0,61	0,64	0,50	0,71	0,78	0,83
	(0,29)	(0,23)	(0,20)	(0,20)	(0,30)	(0,28)	(0,31)	(0,26)	(0,17)
FeCO_2_projection	0,70	0,86	0,79	0,66	0,73	0,63	0,55	0,51	0,42
	(0,66)	(0,13)	(0,31)	(0,34)	(0,17)	(0,28)	(0,18)	(0,31)	(0,26)
HRprojection	0,76	0,58	0,86	0,92	0,75	0,87	0,82	0,69	0,84
	(0,07)	(0,62)	(0,32)	(0,03)	(0,20)	(0,14)	(0,21)	(0,30)	(0,15)

^a^, statistically significant differences among dietary supplementations; *PC* principal component, *VE* ventilation, *FeO2* expired fraction of oxygen, *FeCO2* expired fraction of carbon dioxide, *HR* heart rate

### Physiological and performance variables

**Fig. 1:**
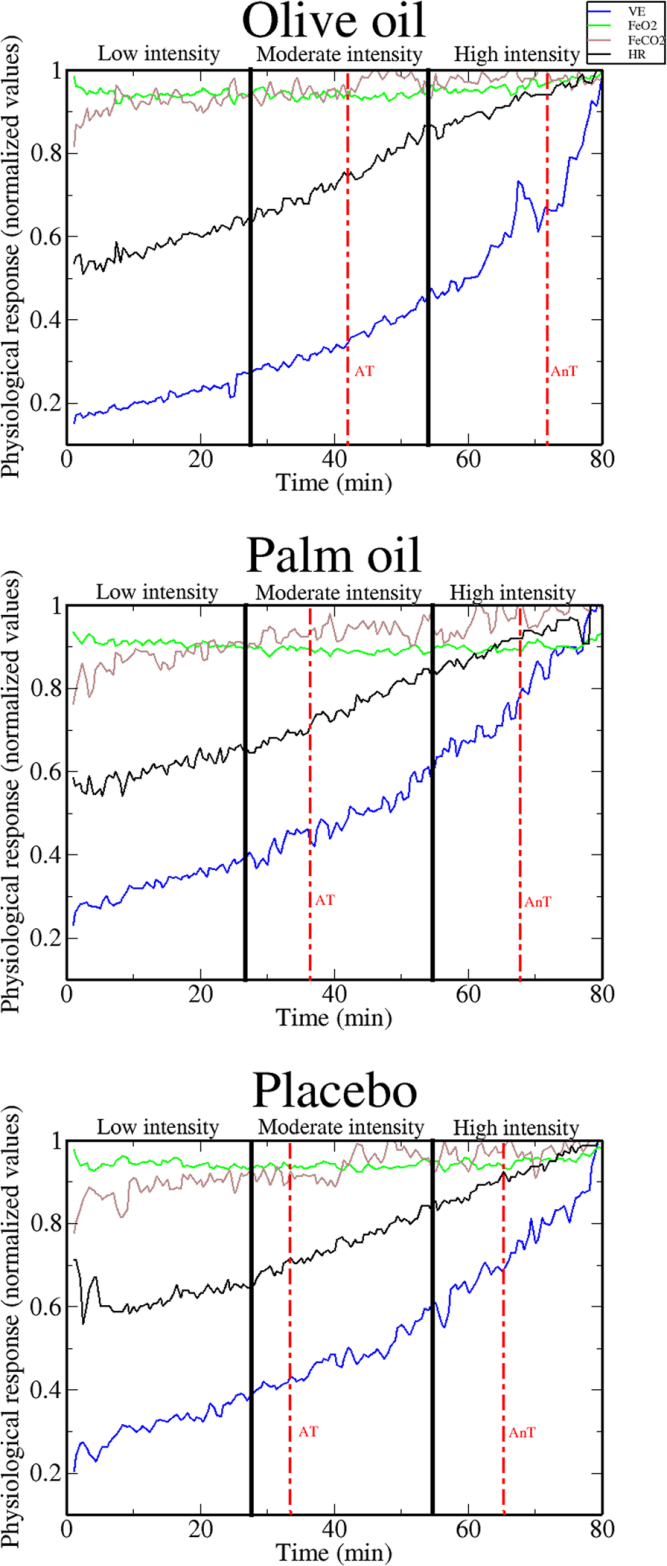
Representation of the selected physiological variables in one single participant during the three dietary supplementations (olive oil, palm oil, and placebo). Each test was divided into three equal sections (low, moderate, and high intensity). The vertical red doted lines indicate the occurrence of aerobic and anaerobic thresholds. Note that a reduction in VE was observed at moderate intensity under olive oil supplementation. AT, aerobic threshold; AnT, anaerobic threshold

Table 3 shows AT (corresponding to moderate exercise intensity), AnT (corresponding to high exercise intensity), and maximal values of cardiorespiratory and performance (i.e., exercising time) variables in the three dietary supplementations. Non-statistically significant differences were found among supplementations. However, while specifically comparing VE, PETO_2_, and PETCO_2_ values corresponding to AT, olive oil revealed significantly lower (VE and PETO_2_) and significantly higher (PETCO_2_) values compared to palm oil supplementation (see Table 3 and Fig. 1).

**Table 3:** Means (standard deviations) of performance and physiological variables, under olive oil, palm oil, and placebo supplementations

Variables	Values	Olive oil	Palm oil	Placebo	Statistical differences
Exercising time (min)	Maximal	77.3 (2.0)	76.3 (2.3)	77.4 (2.3)	
Respiratory rate (b·min^− 1^)	AT	31.6 (1.1)	31.4 (1.8)	32.7 (1.6)	
	AnT	37.5 (3.1)	41.2 (4.1)	39.2 (2.5)	
	Maximal	46.5 (4.1)	52.3 (7.6)	48.7 (5.1)	
Ventilation (L·min^−1^)	AT	53.3 (4.6)	58.7 (4.7)	57.4 (5.5)	(1) *p* = .03; *d* = 0.80
	AnT	83.9 (8.3)	87.7 (10.5)	90.5 (8.7)	
	Maximal	112.9 (9.1)	119.8 (16.5)	117.9 (11.7)	
Oxygen uptake (L·min^−1^)	AT	2.15 (0.16)	2.31 (0.20)	2.36 (0.24)	
	AnT	3.19 (0.2)	3.22 (0.3)	3.40 (0.2)	
	Peak	3.85 (0.1)	3.96 (0.2)	4.00 (0.2)	
PETO_2_ (mmHg)	AT	100.9 (1.3)	102.7 (1.3)	102.3 (1.2)	(1) *p* = .02; *d* = 1.38
	AnT	103.8 (2.2)	105.3 (2.7)	104.2 (1.9)	
	Maximal	108.6 (2.7)	109.6 (3.5)	108.8 (2.8)	
PETCO_2_ (mmHg)	AT	42.1 (0.9)	40.1 (0.8)	40.9 (0.9)	(2) *p* = .02; *d* = 0.14
	AnT	41.7 (3.7)	39.7 (3.8)	41.2 (3.6)	
	Maximal	38.8 (1.7)	37.6 (2.3)	39.4 (1.6)	
FeO_2_ (%)	AT	16.2 (0.1)	16.4 (0.1)	16.2 (0.1)	
	AnT	16.4 (0.2)	16.5 (0.3)	16.4 (0.2)	
	Maximal	16.8 (0.3)	16.8 (0.3)	16.8 (0.3)	
FeCO_2_ (%)	AT	4.3 (0.1)	4.1 (0.1)	4.3 (0.1)	
	AnT	4.4 (0.1)	4.2 (0.2)	4.3 (0.2)	
	Maximal	4.1 (0.2)	4.1 (0.2)	4.3 (0.2)	

*AT* aerobic threshold (moderate exercise intensity), *AnT* anaerobic threshold (high exercise intensity), *PETO*_*2*_, end-tidal partial pressure of oxygen, *PETCO*_*2*_ end-tidal partial pressure of carbon dioxide, *FeO*_*2*_ expired fraction of O_2_, *FeCO*_*2*_ expired fraction of CO_2_, (1), statistically significant differences between olive oil and palm oil; (2), statistically significant differences between olive oil and placebo

## Discussion

The present research was conceived to assess the effect of an acute fatty acid supplementation, in the form of extra virgin olive oil rich in polyphenols, on CRC and performance, compared to palm oil and placebo. An increase in CRC under olive oil supplementation, probably provoked by its high content in polyphenols, was observed. However, these improvements were not reflected on the commonly evaluated maximal performance and physiological variables. These results suggest that CRC could be a sensitive tool to detect systemic effects linked to dietary supplementations.

The improvement in CRC under olive oil supplementation was solely observed at moderate intensity during the incremental test. Specifically, PC_1_ eigenvalues were significantly higher compared to palm oil and placebo supplementations. Since PC_1_ eigenvalues show the ratio of explained variance by PC_1_ [10], these results, indicating an increase in the degree of co-variation among the selected physiological variables, informed about an improvement in CRC. In other words, under olive oil supplementation, the ventilatory efficiency of the cardiorespiratory system seemed larger. These results might be explained by the high content of olive oil in phenolic compounds, tocopherol, or carotenoids, which have been shown to possess antioxidant and anti-inflammatory properties, by producing beneficial effects on lipid oxidation and in general oxidative stress [4, 5]. As pointed out by Sallam and Laher [24], acute bouts of exercise provoke transient damage to contracting skeletal muscles, triggering an inflammatory response that increases the levels of proinflammatory cytokines and reactive oxygen species (ROS) production. Thus, olive oil supplementation could have increased the antioxidant capacity while performing at moderate exercise intensity, reducing the negative effects of ROS accumulation [25]. A feasible explanation for the absence of differences in CRC among dietary supplementations at low and high intensity, might stem from the fact that markers of lipid peroxidation seem to be lower during mild and high intensity compared to moderate exercise [26]. The concentration of lipid peroxidation markers at low and high intensities was not probably enough to impair antioxidant capacity and, therefore, ROS clearance did not occur at a high enough rate to affect cardio-respiratory function. As a result, the antioxidant and anti-inflammatory effects of olive oil could be detected through CRC solely during moderate intensity exercise. Another explanation for the lack of differences at low and high intensity might be related to the Fat_max_ zone [27], which seems to range from 40 to 75% VO_2max_ [28]. The cutting points delimiting the moderate intensity interval in the current study were located at 40.29 + 10.57 and 70.81 + 7.08% VO_2peak_. Thus, the previously observed slight effect of olive oil on fat oxidation [7], could have been magnified at moderate intensity (i.e., Fat_max_), and this might have been detected by CRC analysis.

As depicted in Table 2, while comparing the projections of the selected cardiorespiratory variables onto PC_1_ at moderate intensity, VE projection was shown to be significantly higher in olive oil compared to palm oil supplementation. This means that the increment in the degree of CRC observed under olive oil supplementation was mainly provoked by a change in VE behaviour (i.e., a decrease in the absolute values of VE while performing at moderate intensity and at AT; see Fig. 1 and Table 2), which subsequently led to an increase in co-variation between VE and the other cardiorespiratory variables. The decreased VE under olive oil supplementation could be related to the high content in polyphenols [5], specifically, hydroxytyrosol [29], which may have some peripheral effects on mitochondrial function. As detailed in Hao et al., [29] relatively low doses of hydroxytyrosol increase the expression of all mitochondrial respiratory chain complexes, including ATP synthase, and stimulates mitochondrial biosynthesis pathway. This fact could let to an improvement of central control of VE and, thus, a reduction in ventilatory demands and an increase in ventilatory efficiency (i.e., a reduced VE for the same workload). In contrast, since palm oil and placebo supplementations contained no polyphenols [30], participants probably presented higher ventilatory demands. Accordingly, at moderate intensity (i.e., at AT), PETO_2_ was lower and PETCO_2_ was higher under olive oil, compared to palm oil supplementation (Table 3). Although VE and PETO_2_ were lower and PETCO_2_ was higher under olive oil, compared to both palm oil and placebo supplementations at moderate intensity, the reduced sample size of this study can probably explain why statistically significant differences were only found between olive oil and palm oil supplementations, but not with respect to placebo conditions.

In agreement with Boss et al. [7] and Capó et al. [6, 8], athletic performance (measured through exercising time) was not altered by olive oil supplementation, probably because the improvement on CRC observed in the current study was not strong enough to positively affect performance. Since changes associated to olive oil supplementation seem to be not only happening at cellular or subcellular level but also at systemic levels, and given that CRC is a coordinative variable which might be more sensitive to exercise and dietary supplementations than commonly registered physiological and performance variables, the tracking of changes in CRC may contribute to shed light on other unclear questions regarding the effectiveness of different fatty acid supplementations, such as omega-3 polyunsaturated fatty acids [31] or conjugated linoleic acid [32]. Further research should be conducted to provide an accurate picture of the effectiveness of some controversial dietary supplementations throughout CRC evaluation, such as the physiological mechanisms underlying the impact of beetroot juice supplementation on cardiorespiratory function, in highly-trained endurance athletes [33].

Our findings should be discussed in the light of our methodological limitations. First, oxygen and CO_2_ content and air flow rate were monitored using a low frequency (i.e., every 30 s), and continuous blood pressure monitoring could not be provided in this study. However, the findings obtained in the current research (i.e., high sensitivity and responsiveness of CRC, number of PCs, PC projections) are in full accordance and reinforce those published previously, where respiratory gas exchange was recorded breath by breath and blood pressure was monitored continuously [10, 11]. This means that CRC might be also studied using lower sampling rates when tests have longer duration and provide enough data sets. Second, since inflammatory and lipid peroxidation markers could not be assessed in this research, we cannot guarantee that the changes observed in CRC under olive oil supplementation were provoked by physiological adjustments at this level. Therefore, further research is warranted analysing CRC together with inflammatory and oxidation markers to confirm this hypothesis.

## Conclusions

In conclusion, the supplementation with 25 ml of extra virgin olive oil increased CRC during a progressive walking test while being performed at moderate intensity, although it did not change performance and other physiological markers. CRC analysis appears as a sensitive tool to investigate the physiological and performance effects of dietary supplementations.

## Abbreviations

AnT: Anaerobic threshold
AT: Aerobic threshold
CRC: Cardiorespiratory coordination
FeCO_2_: Expired fraction of CO_2_
FeO_2_: Expired fraction of O_2_
HR: Heart rate
PC_1_: First principal component
PCA: Principal components analysis
PETCO_2_: End-tidal partial pressure of carbon dioxide
PETO_2_: End-tidal partial pressure of oxygen
ROS: Reactive oxygen species
VE: Ventilation
VO_2max_: Maximal oxygen uptake
VO_2peak_: Peak oxygen uptake
